# Fetal exposure to toxic metals (mercury, cadmium, lead, and arsenic) via intrauterine blood transfusions

**DOI:** 10.1038/s41390-024-03504-w

**Published:** 2024-08-30

**Authors:** Iman Al-Saleh, Hissah Alnuwaysir, Reem Al-Rouqi, Hesham Aldhalaan, Maha Tulbah

**Affiliations:** 1 Environmental Health Program, King Faisal Specialist Hospital and Research Centre, Riyadh, 11211 Saudi Arabia; 2 Center for Autism Research, King Faisal Specialist Hospital and Research Centre, Riyadh, 11211 Saudi Arabia; 3 Maternal-Fetal Medicine, Obstetrics & Gynecology Department, King Faisal Specialist Hospital and Research Centre, Riyadh, 11211 Saudi Arabia

## Abstract

**Background:**

Intrauterine blood transfusions (IUBTs) are critical for treating fetal anemia but may expose fetuses to toxic metals. This study assessed mercury (Hg), cadmium (Cd), lead (Pb), and arsenic (As) levels in red blood cell (RBC) transfusion bags used during pregnancy, examined metal exposure in maternal and cord blood, and evaluated fetal health risks.

**Methods:**

Thirty pregnant women who underwent intrauterine blood IUBTs were enrolled in this study. Metal concentrations were measured in one to nine transfusion bags for each participant. These bags contained 8–103 mL volumes and were administered between gestational weeks 18 and 35. We also tested the mothers’ blood for metal levels in the final stages of pregnancy and the umbilical cord blood at birth. The assessment utilized the intravenous reference dose (IVRfD) and the hazard index (HI) to evaluate the non-carcinogenic health risks these metals might pose to the fetus.

**Results:**

Metals were detectable in almost all transfusion bags. The IVRfD was exceeded for Hg in 16 fetuses, Cd in 8 fetuses, Pb in 30 fetuses, and As in 1 fetus. Significant correlations were found between the concentrations of Hg, Cd, and As in transfused RBCs and cord blood. No correlations were observed between these concentrations and maternal blood levels, except for Cd. The influence of multiple IUBTs was positively associated only with Cd levels in the cord (ß = 0.529, 95% confidence intervals (CI) between 0.180 and 0.879). The HI exceeded 1, indicating significant health risks, predominantly from Pb, followed by Hg and Cd.

**Conclusion:**

The findings of this study highlight the significant risk of fetal exposure to toxic metals, mainly Pb, through IUBTs. This underscores the critical need for prescreening blood donors for toxic metals to minimize the potential for long-term adverse effects on the fetus. The research stresses the necessity of balancing the immediate benefits of IUBTs against the risks of toxic metal exposure, underscoring the importance of safeguarding fetal health through improved screening practices.

**Impact:**

- This study highlights the risk of toxic metal exposure through IUBTs, a treatment for fetal anemia.
- Hg, Cd, Pb, and As levels were measured in transfusion bags and linked to fetal exposure through maternal and umbilical cord blood analysis.
- The HI indicates significant Pb exposure risks, underscoring the need for mandatory blood donor screening.
- Recommendations include shifting toward safer practices in managing fetal anemia to protect fetal health.

## Introduction

An intrauterine blood transfusion (IUBT) is administered to fetuses suffering from anemia (a low red blood cell (RBC) count), predominantly due to Rhesus (Rh) blood group incompatibility between the mother and the fetus.^1,2^ While blood banks routinely screen blood for hazardous pathogens,^3^ potential contamination with pollutants, especially toxic metals like cadmium (Cd), lead (Pb), arsenic (As), and mercury (Hg), has been somewhat overlooked. Several studies have highlighted that blood transfusions could serve as a source of exposure to heavy metals and other pollutants, posing a risk to sensitive recipients such as fetuses, premature infants, and neonates.^4–8^ Our recent research indicates that RBC transfusions may expose preterm neonates, especially those requiring multiple transfusions during their Neonatal Intensive Care Unit stay, to Pb and Hg.^9^ Consequently, some experts have advocated for screening transfusion blood for Pb because of its neurotoxic effects.

A study by Gehrie et al.^10^ noted that transfused RBCs could contain Pb concentrations more than 20 times higher than normal, posing a significant risk of pediatric Pb exposure. While Germany’s reference value for blood Pb was 7 µg/dL,^11,12^ the Human Biomonitoring Commission of the German Environment Agency discontinued this reference in 2010, citing Pb’s neurotoxicity and its classification as possibly carcinogenic to humans.^13^ Scientific evidence confirms that Pb levels below 3 µg/dL can impair cognitive function and induce maladaptive behavior in humans and animal models.^14^ Except for the study by Flack et al.,^15^ which reported substantial in utero exposure to Hg and Pb as early as 20 weeks of gestation following an IUBT procedure, further research in this area is scarce.

The current research aimed to evaluate the extent of fetal exposure to toxic metals through IUBTs by analyzing the residual blood from each transfusion bag received by the fetus throughout pregnancy. We directly measured fetal exposure to toxic metals in cord blood at birth and indirectly assessed it through maternal blood samples collected during the third trimester.

## Research design and methodology

A prospective study was conducted from June 2020 to 2023 involving 30 pregnant women who underwent IUBTs at the Maternal-Fetal Therapy Unit, Obstetrics and Gynecology Clinic of the King Faisal Specialist Hospital and Research Centre (KFSH&RC). The indication for IUBTs primarily involved severe fetal anemia due to Rh alloimmunization, diagnosed through regular monitoring antibody titers and Doppler ultrasound measurements of the middle cerebral artery peak systolic velocity (MCA-PSV). Elevated MCA-PSV values and hydrops fetalis observed on ultrasound are key indicators.

The transfused blood comprised packed red blood cells leuco-reduced and irradiated, O Rh D-negative, or cross-matched against the mother’s blood. It was obtained from cytomegalovirus-negative multiple donors and collected within 72 h of the procedure.

The volume of blood administered during IUBTs was determined on the basis of the estimated fetal weight and pre-transfusion hemoglobin level.

The gestational age at the time of IUBTs was calculated on the basis of the last menstrual period and expressed in weeks and days to ensure precise timing, as some fetuses received transfusions within a narrow gestational age span. During the third trimester (between 27 weeks and 6 days to 36 weeks), venous blood samples were collected from the study participants, and cord blood samples were obtained at delivery. Additionally, residual RBCs from the transfusion bags were collected. This study was approved by the Research Ethics Committee of the KFSH&RC (approval number RAC #2200007), and informed consent was obtained from each participant.

We collected 30 venous blood samples, 21 cord blood samples, and residual RBCs from 30 transfusion bags. The number of RBC transfusions received by the participants ranged from 1 to 9 during pregnancy. Residual RBCs from all transfusion bags were collected for 19 patients. However, for 11 patients, one or two bags were missed due to actions beyond our control, dependent on the attending nurse.

Eligibility criteria for participation included being 18 years or older, having a singleton pregnancy, planning to deliver at the KFSH&RC, residing in Riyadh for at least 1 year, and obtaining signed consent forms from both parents. Exclusion criteria encompassed significant chronic diseases such as hypertension, diabetes, autoimmune disorders, and cardiac or renal issues, and women who developed preeclampsia during the study.

### Collection and analysis of blood samples

Venous blood samples (5 mL) were collected from all pregnant women during the third trimester (between 27 weeks and 6 days to 36 weeks), and 4 mL of cord blood was obtained at delivery using ethylenediaminetetraacetic acid (EDTA) tubes (Becton Dickinson, Franklin Lakes, New Jersey). A 200-µL portion of whole blood was then stored in 1.5 mL cryogenic vials (Corning Incorporated, NY) at −30 °C for later analysis of Hg, Cd, Pb, and As levels. Additionally, residual blood from each transfusion bag was collected post-RBC transfusion to assess concentrations of Hg, Cd, Pb, and As.

Each blood/RBC sample (50 µL) was diluted 50× with a specific diluent and measured using inductively coupled plasma mass spectrometry (ICP-MS) (Perkin Elmer NexION®, 2000). Calibration curves for Hg, Cd, Pb, and As in blood showed linearity (*R*^2^ > 0.9994) across the range of 0.25–4.0 µg/L. Accuracy was validated using reference materials and spiked samples, achieving recoveries between 100 and 104.3% with relative standard deviations below 5%. ClinChek controls matched certified ranges. Method detection limits (MDLs) were determined as 0.002 µg/L, 0.044 µg/L, 0.0014 µg/L, and 0.0022 µg/L for Cd, Pb, As, and Hg, respectively. Complete method details can be found in the Supplementary Material.

### Intravenous reference dose calculation

To enable the safety of toxic metal exposure to be calculated from blood transfusions, the intravenous reference dose (IVRfD) was calculated for each metal, following the procedures described in previous studies.^15–17^ Determining the IVRfD was crucial because it enabled the study to evaluate the potential health risks posed by the Hg, Cd, Pb, and As concentrations observed in RBC transfusion bags. This approach facilitated an informed understanding of the risks associated with exposure to metals during blood transfusions.

### Risk assessment

We calculated the hazard index (HI) to estimate the potential non-cancer adverse health effects of toxic metals by summing the IVRfD values of each metal detected in the RBC transfusion bag.^18^ An HI value ≤1 indicates that the exposure is unlikely to result in non-cancer adverse health effects.^18^

### Statistical analysis

Values within the text are presented as means, medians, minimums, and maximums. The relationship between the metal contents across different matrices was assessed using the Spearman rank correlation coefficient. Moreover, differences in metal concentrations between cord and maternal blood were analyzed using the Mann–Whitney U test. β regression coefficients and 95% confidence intervals (95% CIs) were calculated to evaluate the impact of the total number of IUBTs and their timing within the gestational period. All values below the MDL were substituted with half of the MDL value. Statistical analyses were performed using SPSS software version 20 for Windows (IBM Corp., Armonk), with a significance level set at *p* < 0.05.

## Results

A total of 30 women between 30 and 46 years received 1–9 IUBTs per fetus to treat fetal anemia secondary to Rh isoimmunization. The volume of each IUBT ranged from 8 to 103 mL and was administered on the basis of fetal weight, gestational age, pre-transfusion hemoglobin levels, hemoglobin drop between transfusions, and the presence of hydrops fetalis. The gestational age at the time of IUBTs ranged from 18 to 35 weeks. The median (range) pre-, mid-, and post-transfusion hemoglobin levels were 6.3 (1.2–14), 11.8 (2.8–17.7), and 14.6 (7.1–18.2) g/dL, respectively. Estimated fetal weight was not measured for all participants. Out of 141 instances of IUBT, fetal weight was recorded for only 37 cases, showing a median of 1450 g (range: 324–2654 g). These measurements exhibited a strong correlation (*r* = 0.959, *p* < 0.001) with the standard World Health Organization fetal weight chart for gestational age.^19^ Table 1 summarizes RBC transfusion parameters, demographic data, and toxic metal concentrations for all patients. Furthermore, patient-specific data are detailed in the Supplementary Material (Supplementary Table S1 and Supplementary Table S2).

**Table 1:** Transfusion information, fetus demographic, concentration, and IVRfD of metals in RBC transfusions, maternal venous blood, and cord blood.

Stage		Unit	*N*	Mean	Median	Minimum	Maximum
RBC transfusion	Number of IUBT		144	3.30	3.00	1	9
	Gestational age	Weeks and days	144	28 weeks and 4 days	28 weeks and 5 days	18.0	35.0
	Transfusion volume	mL	138	49.43	50.00	8	103
	Estimated fetal weight	gm	37	1507.62	1450.00	324	2654
	Pre-transfusion hemoglobin	g/dL	136	8.39	8.40	1.20	16.00
	Mid-transfusion hemoglobin	g/dL	108	13.68	14.45	2.80	18.80
	Post-transfusion hemoglobin	g/dL	128	15.43	16.00	7.10	19.00
	Hg	µg/L	127	1.499	0.739	0.0011	28.225
	Cd	µg/L	127	0.910	0.715	0.001	3.947
	Pb	µg/L	127	14.640	12.812	3.116	41.439
	As	µg/L	127	0.808	0.483	0.0007	8.423
	IVRfD						
	Hg	µg/kg	125^a^	0.0766	0.0319	0.000	2.117
	Cd	µg/kg	125	0.0414	0.0271	0.00004	0.174
	Pb	µg/kg	125	0.672	0.543	0.065	2.763
	As	µg/kg	125	0.0365	0.0204	0.000023	0.430
Third trimester-venous blood	Hg	µg/L	30	0.399	0.334	0.0011	1.075
	Cd	µg/L	30	0.851	0.281	0.001	3.526
	Pb	µg/L	30	4.954	4.254	0.720	19.566
	As	µg/L	30	0.413	0.240	0.0007	1.859
Delivery-cord blood	Hg	µg/L	21	0.680	0.515	0.051	2.029
	Cd	µg/L	21	0.350	0.079	0.001	2.371
	Pb	µg/L	21	4.273	4.352	0.233	12.053
	As	µg/L	21	0.377	0.093	0.0007	3.558

^a^The discrepancy in the total number was due to two cases with missing transfusion volumes.

### Concentration of toxic metals in RBC transfusion bags

We analyzed 127 RBC transfusion bags and found that 98% of the bags contained detectable levels of Hg, 90% contained such levels of Cd and As, and 100% contained such levels of Pb. The median concentrations were as follows: Hg = 0.739 µg/L, Cd = 0.715 µg/L, Pb = 12.812 µg/L, and As = 0.483 µg/L. Furthermore, three bags were exposed to Hg levels above the United States Environmental Protection Agency (U.S. EPA) reference value of 5.8 µg/L,^20^ and eight bags were found to be exposed to levels over 3.5 µg/L.^21^ Although no Cd levels were found to surpass the U.S. Occupational Safety and Health Administration (OSHA)’s threshold of 5 µg/L,^20,22^ 51 bags were found to exceed 1 µg/L, the typical level found in smokers,^23^ For Pb, while no samples surpassed the U.S. Centers for Disease Control and Prevention (CDC)’s action level of 50 µg/L for children,^24^ four bags contained more than 35 µg/L,^25^ 91 bags exceeded the recommended threshold of 10 µg/L for donor RBC units intended for pediatric use,^10^ and 125 bags exceeded the 5 µg/L interim reference level.^26^ For As, while the median concentration was below the level found in a previous study,^9^ 18 bags exceeded the 1.4 µg/L threshold for children, while 10 surpassed the 2 µg/L threshold for adults.^27^

### IVRfD assessment of toxic metals

**Fig. 1:**
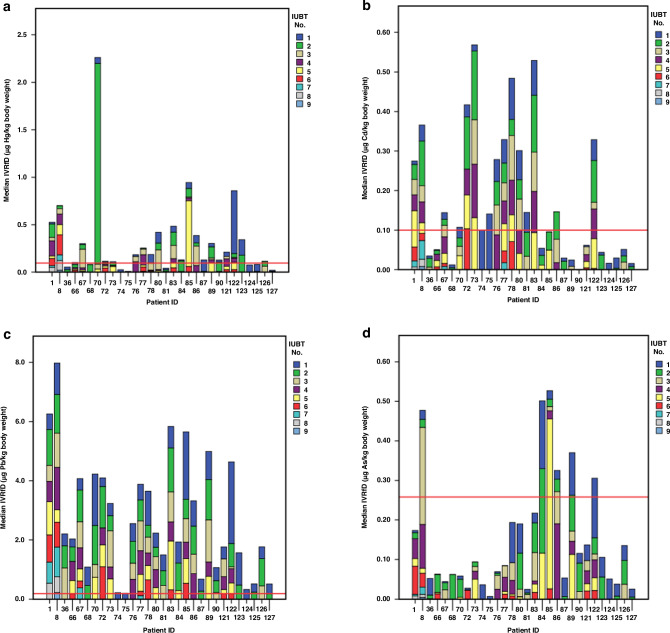
Assessment of heavy metal exposure in pregnant women. This figure presents stacked bar charts depicting the IVRfD for heavy metals in pregnant women undergoing multiple IUBTs. Panel (**a**) displays the data for Hg, panel (**b**) for Cd, panel (**c**) for Pb, and panel (**d**) for As. Red lines across each panel indicate the permissible IVRfD levels in µg/kg body weight, set at 0.095 for Hg, 0.1 for Cd, 0.19 for Pb, and 0.258 for As.

Figure 1a–d illustrates the IVRfD of four metals to which fetuses are exposed during an IUBT. Each stacked bar represents the cumulative exposure for each fetus, corresponding to the IVRfD of each metal. The median IVRfD values for Hg, Cd, Pb, and As were 0.0319, 0.0271, 0.0543, and 0.0204 µg/kg, respectively. For Hg, although the median IVRfD was below the aforementioned level of 0.07 µg/kg,^15^ 16 fetuses—aged from 19 weeks and 3 days to 33 weeks and 6 days—experienced Hg exposure above the IVRfD threshold of 0.095 µg/kg per transfusion.^28^ Notably, one fetus underwent four transfusions while five received two each, with median exposure levels of 0.140 µg/kg (range: 0.098–2.117 µg/kg). Cd exhibited a median IVRfD higher than the typical 0.01 µg/kg for preterm neonates.^28^ Moreover, eight fetuses exceeded the IVRfD benchmark of 0.1 µg/kg per transfusion,^28^ with one fetus undergoing four transfusions and three receiving two each. The median exposure levels were 0.113 µg Cd/kg body weight (range: 0.101–0.174 µg/kg). For Pb, the data indicated that fetuses aged between 18 weeks and 34 weeks and 6 days were subjected to 117 transfusions, with the Pb levels surpassing the 0.19 µg/kg threshold.^15^ Ten fetuses were exposed to 5–9 bags with elevated Pb levels. Finally, we calculated an IVRfD of 0.285 µg/kg body weight per transfusion for As.^28^ based on 95% gastrointestinal absorption.^29^ and an oral reference dose of 0.3 µg/kg body weight per day.^30^ Only one fetus exceeded this IVRfD level as it received a transfusion with 0.285 µg/kg body weight of As.

### Heavy metals in maternal versus cord blood: concentrations and correlations

This study’s findings indicate significant differences in heavy metal concentrations between cord and maternal venous blood. Hg levels were found to be higher in cord blood (median: 0.515 µg/L; range: 0.051–2.029 µg/L) compared with maternal blood (median: 0.334 µg/L; range: 0.0011–1.075 µg/L; *p* = 0.034). Conversely, Cd concentrations were found to be approximately five times higher in maternal blood (median: 0.281 µg/L; range: 0.001–3.526 µg/L) than in cord blood (median: 0.079 µg/L; *p*-value = 0.031). Pb exhibited similar median levels in cord (4.352 µg/L) and maternal blood (4.254 µg/L) with no significant difference. Lastly, As levels were three times higher in maternal blood (median: 0.24 µg/L) than in cord blood (median: 0.093 µg/L; *p*-value = 0.092).

Furthermore, a correlation analysis revealed a strong association between Hg levels in RBC transfusions and cord blood (*r* = 0.803, *p* < 0.001) but not with maternal blood. However, Hg levels in cord and maternal blood were positively correlated (*r* = 0.57, *p* < 0.001). Moreover, moderate correlations were found for Cd levels between RBC transfusions and both types of blood (cord: *r* = 0.48, *p* = 0.032; maternal: *r* = 0.551, *p* < 0.001), with a positive correlation found between cord blood and maternal levels (*r* = 0.51, *p* = 0.022). By contrast, Pb levels displayed no significant correlations across RBC transfusions, cord blood, and maternal blood. Finally, As levels in RBC transfusions were positively correlated with cord blood (*r* = 0.504, *p* = 0.024) but exhibited no significant correlation with maternal venous As or between cord and maternal blood.

### Impact of transfusion frequency and timing on metal levels

Table 2 outlines the impact of the total number of transfusions per mother/fetus dyad and their timing. Our analysis revealed a significantly positive association only between the total number of IUBTs and Cd levels in cord blood (ß = 0.529, 95% CI: 0.180 to 0.879). However, the timing of transfusions did not significantly influence metal levels.

**Table 2:** Impact of the total number of IUBTs and the timing of transfusions throughout the gestational age on metal levels in cord and maternal blood.

Model	Metal (µg/L)	Total number of IUBT			Timing of transfusion		
		ß	95% CI		ß	95% CI	
			Lower 95% CI	Upper 95% CI		Lower 95% CI	Upper 95% CI
1	Cord Hg	−0.943	−2.175	0.288	1.610	−1.006	4.226
	Maternal Hg	−0.030	−0.466	0.406	0.204	−0.722	1.131
2	Cord Cd	**0.529**	**0.180**	**0.879**	−0.840	−1.680	0.000
	Maternal Cd	−0.028	−0.415	0.359	0.087	−0.842	1.017
3	Cord Pb	−0.143	−1.295	1.009	0.182	−2.214	2.578
	Maternal Pb	−0.159	−1.507	1.189	0.759	−2.045	3.563
4	Cord As	−0.272	−0.638	0.094	0.093	−0.690	0.877
	Maternal As	−0.173	−0.787	0.441	0.977	−0.336	2.291

Bold characters denote significant associations (*p* < 0.05).

## Discussion

This study highlights critical concerns regarding fetal exposure to toxic metals through RBC transfusions, aligning with the findings of previous research. The notably high Hg, Cd, Pb, and As detection rates within RBC transfusion bags suggest that fetuses are particularly susceptible to these toxicants during vital developmental stages. This emphasizes the necessity of implementing stringent screening processes to detect and reduce the presence of toxic metals in blood donors before donation, thereby safeguarding fetal health.

Our findings highlight significant variability in Hg exposure levels among fetuses receiving RBC transfusions, with several instances surpassing the US EPA.^20^ and researcher-proposed safety thresholds.^21^ The exceedance of these thresholds, particularly the IVRfD,^28^ raises concerns about potential adverse health effects. The observed median exposure level of 0.140 µg Hg/kg body weight, higher than previously reported,^15^ suggests that certain fetuses are at increased risk, especially those undergoing multiple transfusions. This discrepancy with prior studies can be attributed to variations in transfusion practices or differences in the Hg content of transfused blood. Our results demonstrate a significant transfer of Hg via RBC transfusions into cord blood, suggesting efficient crossing through the placental barrier.^31^ This is supported by the strong correlation between Hg levels in RBC transfusions and cord blood. Additionally, the positive correlation between Hg levels in cord and maternal venous blood underscores a systemic circulation of Hg, typically in an organic form such as methylmercury,^32^ which is known for its high placental transfer efficiency.^33^ This highlights the potential risks to fetal health from Hg exposure during transfusions. Furthermore, the median Hg concentration in cord blood was notably higher than in maternal venous blood, a difference consistently observed in prior studies and has been attributed to the presence of high-affinity fetal-specific serum albumin proteins in the cord blood compared to maternal blood.^34–38^ Nevertheless, the median Hg levels observed in cord (0.51 µg/L) and maternal blood (0.334 µg/L) are lower than those reported in previous studies, specifically in cord blood (1.949 µg/L) and maternal blood (2.876 µg/L) from over a decade ago.^34^ Crucially, none of these levels exceeded the critical threshold value for Hg in blood.^20^

Our research confirms that fetuses are exposed to Hg through RBC transfusions, a matter that raises significant concerns due to the vulnerability of the study population. Given the cumulative nature of Hg, it is critical to minimize early-life exposure to prevent serious health complications. This is particularly important for preventing neurodevelopmental delays that may manifest later in life.^39^

Furthermore, our findings regarding Cd levels in transfusion bags were concerning. While these levels do not exceed OSHA’s highest safety thresholds,^22^ they surpass those typically observed in high-exposure populations such as smokers.^23^ This is particularly alarming given the sensitive developmental stage of the fetuses. Moreover, the median IVRfD for Cd was higher than previously reported levels for preterm neonates, indicating a potential increase in the risk of adverse health outcomes in affected fetuses.^28^ The fact that eight fetuses experienced Cd exposures above the established IVRfD benchmark underscores the critical need for reevaluating safety thresholds and monitoring practices in transfusions for this population. Additionally, our findings suggest a transfer of Cd between maternal and fetal circulations, corroborated by the correlations observed between RBC transfusions and cord and maternal blood. The detectable presence of Cd in maternal and cord blood supports Cd’s ability to cross the placental barrier; however, the significantly higher maternal Cd concentration suggests that the placenta acts as a partial barrier.^34,40^ This aligns with research demonstrating reduced Cd levels in cord blood, although the present study confirms that median Cd levels in cord and maternal venous blood were significantly lower than the 0.704 and 0.983 µg/L reported in Saudi Arabia.^34^ Despite these generally lower levels, elevated Cd levels (>1 µg/L) were observed in 11 maternal venous and three cord blood samples. This elevation is likely to be related to tobacco exposure,^23^ including one case of maternal smoking and 14 cases of exposure to second-hand smoke. Our data indicate that RBC transfusions could expose fetuses to Cd during a critical developmental period. Moreover, given Cd’s long half-life (10–30 years) and its tendency to accumulate in tissues, a heightened potential exists for long-term health risks, including cancer.^41^

While we found no transfusion bags that surpassed the CDC’s action level for Pb exposure in children,^24^ several bags exceeded less stringent thresholds.^25,26^ Specifically, four bags contained lead levels higher than a cutoff value proposed by the CDC’s subcommittee on Pb poisoning prevention; additionally, a significant number exceeded the interim reference level designated for evaluating potential adverse effects of dietary Pb in children and women of childbearing age.^26^ In our analysis, we observed that fetuses that received RBC transfusions between 18 and almost 35 weeks of gestation were exposed to Pb levels that exceeded the safety threshold of 0.19 µg/kg body weight in a significant number of cases.^15^ Specifically, 117 transfusions delivered Pb doses above this critical level. Furthermore, a subset of 10 fetuses experienced exposure from 5–9 transfusion bags, each of which contained elevated Pb levels. This exposure pattern underscores the urgent need to address and mitigate Pb risks in medical interventions, particularly during the critical stages of fetal development. Given Pb’s neurotoxicity and classification as probably carcinogenic in humans,^13^ the German Federal Agency’s Commission suspended the reference level for blood Pb,^9^ suggesting that an IVRfD of 0.19 µg Pb/kg body weight cannot be considered acceptable. Moreover, the median Pb concentration in cord blood was not significantly different from that in maternal venous blood, consistent with findings from our previous study that reported lower Pb levels in both blood types.^34^ In the current study, of the 21 cord and 30 maternal blood samples analyzed, 6 and 9 samples exceeded the 50 µg/L Pb threshold, respectively.^24^ Although we found no direct correlation between Pb levels in transfusion bags and those in blood samples, one cannot ignore the potential for RBC transfusions to contribute to Pb exposure. This concern is heightened by the high environmental Pb levels to which the Saudi population is exposed from typical as well as unusual sources,^42–44^ which have been further amplified following recent increases in industrialization and urbanization.^45^ Given the significant risks associated with cumulative Pb exposure, particularly to the developing brains and organs of vulnerable populations, this is an area of serious concern.^46^

In this study, the median As concentration in RBC transfusion bags was found to be notably lower than concentrations previously reported in transfusions given to preterm neonates.^9^ Despite this, our findings contrast with those of other research that detected no arsenic in blood transfusions,^23^ indicating that As contamination can vary significantly between different clinical settings. Crucially, several transfusion bags exceeded the recognized safety thresholds for children and adults,^27^ highlighting a sporadic but significant risk of higher As exposure through transfusions. Although RBC transfusions are not typically a significant source of As exposure, the observed positive correlation between As levels in RBC transfusions and cord blood is noteworthy. As such a correlation was absent between maternal venous blood and RBC transfusions and cord blood, there is a need to further investigate the mechanisms of As transfer and its accumulation in the fetus compared with the mother. Additionally, elevated As levels above the RV95 threshold for children.^27^ were detected in three cord and seven maternal blood samples. However, caution is required when interpreting these results, as our measurements were of total As rather than its more toxic inorganic forms or urinary methylated metabolites, which could differ significantly in terms of health implications.^47^

When examining the impact of multiple IUBTs and fetal gestational age at transfusion on metal levels in cord and maternal blood, we observed that fetuses receiving more frequent transfusions may experience higher exposure to Cd, a potentially harmful metal. This finding suggests a need for further investigation into sources and potential health effects of Cd exposure during multiple blood transfusions in fetuses. The lack of significant associations for other metals in cord blood may be due to a range of other factors not considered in this analysis, such as differences in the efficiency of placental transfer of metals, genetic variability among mothers and fetuses that might influence metal metabolism, accumulation dynamics, and the limited sample size of the study. Further research is necessary to explore these potential influences to better understand the dynamics of metal exposure during pregnancy.

Our study revealed that 17 fetuses showed HI > 1, with Pb being the major contributor, followed by Hg and Cd. This indicates that exposure to a combination of these toxic metals through RBC transfusions at such a vulnerable age is concerning, suggesting an increased potential for overall non-cancer adverse health effects. Such effects include those on neurodevelopment, obesity, congenital anomalies, and cardiometabolic diseases, which might be reflected in children’s early or later stages of life.^9,48–50^

Our study has some limitations that warrant attention. (1) The small sample size may have constrained our ability to identify correlations among certain metals. (2) There is a lack of essential data related to RBC transfusions. (3) The estimated fetal weight was not consistently measured or recorded in patient charts. (4) Not all RBC transfusions were analyzed for metal content due to oversight by nursing staff in keeping the remaining residual. (5) Cord blood samples were not obtained for all participants in the delivery room. (6) Pre-transfusion heavy metal levels in the mothers were not measured, which may have contributed to the heavy metal levels observed in the cord blood samples. (6) The HI did not consider the combined effects of the metals as a mixture. Consequently, the potential health hazard may be underestimated if the interactions between the metals are synergistic or overestimated if these interactions are antagonistic.^51^

In conclusion, our study’s findings significantly enhance the understanding of fetal exposure to toxic metals during IUBT procedures. They also contribute valuable insights to the limited research that exists in this field. Furthermore, this study’s findings demonstrate that Hg, Cd, Pb, and As levels often exceed established safety thresholds, which highlights not only the efficient transfer of these metals through the placental barrier but also the risks of cumulative exposure associated with multiple transfusions. While IUBTs are essential for treating fetal anemia, they may have significant health consequences later in life, particularly with regard to neurodevelopment. These findings underscore the urgent need to reevaluate blood donor screening protocols and develop revised clinical guidelines to reduce fetal metal exposure and enhance fetal health. Additionally, long-term monitoring must be initiated for children who have undergone an IUBT.

## Data Availability

Data are available upon request.

## Supplemental information

Supplemental Material
Supplemental Table
