# Supplementary material for: Fetal exposure to toxic metals (mercury, cadmium, lead, and arsenic) via intrauterine blood transfusions
Source: Pediatr Res. 2024 Aug 30;97(2):647–54. doi: 10.1038/s41390-024-03504-w (PMC12014473; doi:10.1038/s41390-024-03504-w)
Supplement: Supplementary file 1 — Supplemental Material [file 41390_2024_3504_MOESM1_ESM.pdf]

# Fetal exposure to toxic metals (mercury, cadmium, lead, and arsenic) via intrauterine blood transfusions

Iman Al-Saleh[1,*], Hissah Alnuwaysir[1], Reem Al-Rouqi[1], Hesham Aldhalaan[2], Maha Tulbah[3]

[1]Environmental Health Program, Obstetrics & Gynecology Department, King Faisal Specialist Hospital and Research Centre, P.O. Box: 3354, Riyadh 11211, Saudi Arabia

[2]Center for Autism Research, Obstetrics & Gynecology Department, King Faisal Specialist Hospital and Research Centre, P.O. Box: 3354, Riyadh 11211, Saudi Arabia

[3]Maternal-Fetal Medicine, Obstetrics & Gynecology Department, King Faisal Specialist Hospital and Research Centre, P.O. Box: 3354, Riyadh 11211, Saudi Arabia

**[*]Corresponding Address:**

Dr. Iman Al-Saleh (MBC#03)

Environmental Health Program, King Faisal Specialist Hospital & Research Centre

P.O. Box: 3354, Riyadh 11211, Saudi Arabia

Phone No.: 00966114424772; E-mail: iman@kfshrc.edu.sa

*Analytical methods*

Each blood/RBC sample (50 µL) was diluted (50×) with a diluent mixture containing 0.5% nitric acid (Fisher Scientific, PA), +0.05% Triton-X (Sigma-Aldrich™, MO), +2% methanol (Fisher Scientific, PA), (all v/v), +250 µg/L gold, and +0.1 µg/L internal standards [rubidium ($^{103}$Rh) for arsenic and iridium ($^{193}$Ir) for Pb, Hg, and Cd]. Metals were measured using inductively coupled plasma–mass spectrometry (ICP–MS; Perkin Elmer NexION®, 2000). The diluent intensity was subtracted from the calibrator standards, quality control, and patient samples. The calibration curves for Hg, Cd, Pb, and As in blood covered a range of 0.25–4.0 µg/L. These ranges were deemed satisfactory, exhibiting linear correlation coefficients as follows: Cd had 0.9997 ± 0.0003 ($N$ = 11), Pb showed 0.9994 ± 0.0006 ($N$ = 11), As demonstrated 0.9998 ± 0.0003 ($N$ = 10), and Hg reported 0.9998 ± 0.0002 ($N$ = 10). The method's accuracy was assessed using external assurance reference materials and internal quality control samples. Pooled blood samples were spiked with 0.75, 1.5, and 3 µg/l metal levels and run in parallel with patient samples to check between-runs precision. The recovery values of the three metal concentrations in blood were as follows: 100, 100.1, and 100.4% (Cd); 101.3, 101.3, and 99.9% (Pb); 104.3, 103.1, and 101.4% (As); and 97.7, 99.6, and 101.9% (Hg). The sample replicates' relative standard deviation (RSD%) was <5%, except for 0.75 µg/l for Pb and Hg, which were 6.5% and 7.5%, respectively.

The within-run precision RSD% values for 10 replicates of spiked pooled blood samples with 0.3 0.75 and 1.5 µg/L were 3.4, 3.2, and 2.0% (Cd); 3.9, 4.6, and 2.6% (Pb); 3.7, 2.4, and 4.1% (As); and 3.6, 2.3, and 3.7% (Hg), respectively.

Three sets of lyophilized standard reference materials, namely, ClinChek controls manufactured by Recipe® Chemicals and Instruments GmbH (Munich, Germany), were analyzed in each run. Our analytical results for Levels I, II, and III in µg/L were 1.93, 4.05, and 7.22 (Cd); 34.68, 87.3, and 239.4 (Pb); 2.83, 9.33, and 18.15, (As); and 3.57, 7.81, and 17.53 (Hg). These findings closely aligned with the certified recommended ranges (in µg/L) for Levels I, II, and III, respectively: Cd (1.18–1.97, 2.83–4.24, and 5.63–8.44); Pb (30.1–45.2, 76.5–115, and 208–312); As (2.42–3.62, 7.66–11.5, and 15.4–23.5); and Hg (2.42–3.62, 7.66–11.5, and 15.4–23.5).

The method detection limit (MDL) was calculated by multiplying the standard deviation (SD) of the 10 replicates of the blank levels with the Student's *t*-value corresponding to $n-1$ degrees of freedom and 99th percentile. The blood's MDLs for Cd, Pb, As, and Hg were 0.002, 0.044, 0.0014, and 0.0022 µg/L, respectively.
